# Role of the vector genome and underlying factor IX mutation in immune responses to AAV gene therapy for hemophilia B

**DOI:** 10.1186/1479-5876-12-25

**Published:** 2014-01-25

**Authors:** Geoffrey L Rogers, Ashley T Martino, Irene Zolotukhin, Hildegund CJ Ertl, Roland W Herzog

**Affiliations:** 1 Department of Pediatrics, Division of Cellular and Molecular Therapy, University of Florida, Gainesville, Florida, USA; 2 The Wistar Institute, Philadelphia, Pennsylvania, USA

**Keywords:** AAV, Gene therapy, Hemophilia B, Factor IX, Immune response

## Abstract

**Background:**

Self-complementary adeno-associated virus (scAAV) vectors have become a desirable vector for therapeutic gene transfer due to their ability to produce greater levels of transgene than single-stranded AAV (ssAAV). However, recent reports have suggested that scAAV vectors are more immunogenic than ssAAV. In this study, we investigated the effects of a self-complementary genome during gene therapy with a therapeutic protein, human factor IX (hF.IX).

**Methods:**

Hemophilia B mice were injected intramuscularly with ss or scAAV1 vectors expressing hF.IX. The outcome of gene transfer was assessed, including transgene expression as well as antibody and CD8^+^ T cell responses to hF.IX.

**Results:**

Self-complementary AAV1 vectors induced similar antibody responses (which eliminated systemic hF.IX expression) but stronger CD8^+^ T cell responses to hF.IX relative to ssAAV1 in mice with *F9* gene deletion. As a result, hF.IX-expressing muscle fibers were effectively eliminated in scAAV-treated mice. In contrast, mice with *F9* nonsense mutation (late stop codon) lacked antibody or T cell responses, thus showing long-term expression regardless of the vector genome.

**Conclusions:**

The nature of the AAV genome can impact the CD8^+^ T cell response to the therapeutic transgene product. In mice with endogenous hF.IX expression, however, this enhanced immunogenicity did not break tolerance to hF.IX, suggesting that the underlying mutation is a more important risk factor for transgene-specific immunity than the molecular form of the AAV genome.

## Background

Hemophilia B is the X-linked monogenetic disorder caused by the loss of functional coagulation factor IX (F.IX), resulting in a deficiency in the ability of blood to clot. In addition to increased propensity for bleeding after trauma or injury, spontaneous bleeds can occur in capillaries, particularly in the joints, resulting in tissue damage over time. Bleeds into critical closed spaces can be life-threatening. Currently, hemophilia B is treated by intravenous administration of F.IX concentrate, either plasma-derived or recombinant, in order to restore hemostasis. Because of the short half-life of the protein in circulation, frequent injections are required to provide prophylaxis or to treat patients with severe disease on demand. Gene therapy represents an attractive alternative to protein replacement therapy, as it would involve a single injection to provide long-term intrinsic production of F.IX.

Among potential gene therapies for hemophilia B, the use of adeno-associated virus (AAV) as a gene delivery vector has shown the most success to date [1]. AAV is a dependovirus, a parvovirus that is unable to replicate in the absence of a helper virus (typically adenovirus). For use as a gene therapy vector, all viral genes are removed, leaving only the inverted terminal repeats required for packaging around the transgenic construct. The various serotypes of AAV have different tropisms, which allow for gene transfer to numerous target tissues [2]. For instance, AAV1 can effectively transduce skeletal muscle, while AAV8 has strong tropism for liver tissue. Pre-clinical studies in animals established that the risk of immune responses to F.IX is substantially affected by the route of vector administration and by the underlying genetic defect. *F9* null mutations (complete absence of protein, for example resulting from a gene deletion) are most likely associated with strong immune response, while mutations preserving some level of endogenous, albeit non-functional F.IX expression, reduce the risk for immune responses [3-6].

Recent clinical trials are based on liver-directed gene transfer. Hepatocytes are the normal site of F.IX synthesis. Furthermore, high levels of antigen expression in hepatocytes promote induction of regulatory T cells, resulting in immune tolerance induction to the transgene product. This approach is even able to reverse an ongoing antibody response against F.IX [4,7,8]. Sustained expression of F.IX by hepatic gene transfer has now been demonstrated in hemophilia B patients, following successes in large animals model, including non-human primates and hemophilia B dogs [9-11].

AAV vectors traditionally contain a single-stranded DNA genome (ssAAV) with a packaging limit of approximately 5 kb. By modifying one of the inverted terminal repeats, it is possible to force the virus to package a self-complementary double-stranded DNA genome (scAAV), thereby bypassing the need to for second-strand synthesis, one of the rate-limiting steps in AAV transduction [12]. A disadvantage of this strategy is the further reduced packaging limit. Nonetheless, scAAV vectors expressing F.IX from liver-specific promoters have been optimized and are currently used in clinical trials [9]. In addition to more rapid transgene expression, scAAV vectors often produce higher transgene levels than ssAAV with an equivalent input dose [11]. At the same time, we found that scAAV vectors elicited stronger innate immune responses in the liver than ssAAV, likely because of enhanced toll-like receptor 9 (TLR9) signaling. Consistent with prior studies by others, hepatic innate immune responses to AAV vectors were dependent on TLR9, an endosomal receptor that recognizes unmethylated CpG DNA motifs [13-15]. In our hepatic gene transfer model, the heightened innate response did not increase adaptive immune responses to the F.IX transgene product but caused modest increases in B and T cell responses to the capsid antigens of the vector.

Skeletal muscle represents an alternative target tissue for AAV-F.IX gene transfer. Upon gene transfer myofibers are capable of producing biologically active material, and the first clinical trial on AAV-F.IX gene transfer utilized intramuscular injections at multiple skeletal muscle sites as the route of vector administration [16-19]. F.IX-expressing muscle fibers may persist in humans for at least 10 years after initial gene transfer [20]. However, a concern about muscle-directed gene transfer is the increased risk of immune responses against F.IX. Hence, in this study we chose the more immunogenic intramuscular route to assess the potential for B and T cell responses against F.IX as a function of the vector genome (scAAV vs ssAAV) and the underlying *F9* gene mutation. The results show a stronger and more destructive CD8^+^ T cell response using scAAV in mice with a *F9* gene deletion, while mice expressing truncated hF.IX remained tolerant to F.IX regardless of vector genome conformation.

## Methods

### Animal strains and experiments

Hemophilia B mice with targeted deletion of murine *F9* (‘HB’) had been bred on C3H/HeJ background for >10 generations [21]. Mice transgenic for truncated hF.IX (human *F9* complementary DNA including a 0.3-kb portion of intron I expressed from liver‒specific transthyretin promoter) were as published [22]. These animals express hF.IX with late stop codon at amino acid residue 338 (‘LS’). This line was originally numbered as LS-37 and contains 6 copies of the hF.IX gene [22]. The line was repeatedly backcrossed onto C3H/HeJ background (>10 generations), and finally crossed with HB mice in order to eliminate endogenous murine F.IX expression [3]. Animals were housed under specific pathogen-free conditions at the University of Florida and treated under Institutional Animal Care and Use Committee-approved protocols. All animals were male and 6–8 weeks old at the onset of the experiments; all cohorts contained at least 4 mice per group.

AAV vectors were administered intramuscularly into two sites: quadriceps and tibialis anterior of one hind limb, as previously described [23]. Plasma samples were collected by tail bleed into citrate buffer as described [21].

### AAV vectors

ssAAV vector expressing human F.IX cDNA (including a 1.4-kb portion of intron I) from the CMV IE enhancer/promoter was as published [19]. For construction of scAAV, the human F.IX coding sequence (lacking intronic or 3′ untranslated sequences) was cloned into an scAAV-CMV-GFP construct, replacing the GFP sequence. This construct contains a small β-globin/IgG chimeric intron. Vector genomes were packaged into AAV serotype 1 capsid by triple transfection of HEK-293 cells. Vector particles were purified by iodixanol gradient centrifugation, and vector titers determined by dot blot hybridization and confirmed by Western blot using a reference standard of known titer for comparison.

### Analysis of plasma samples

Plasma was analyzed for hF.IX expression, anti-hF.IX IgG1, and anti-AAV1 IgG2a by enzyme-linked immunosorbent assay (ELISA) as previously described [13,21]. For the anti-capsid antibody ELISAs, sample wells were coated with 2.5 × 10^9^ vg/well intact AAV1 particles. The assay for anti-hF.IX IgG1 was sensitive to ~200 ng/mL. Anti-hF.IX inhibitory activity was assessed using the Bethesda assay, as previously described [3]. One Bethesda unit (BU) represents the inhibition of 50% of clotting activity. Clotting assays were performed on a STart® Hemostasis Analyzer (Diagnostica Stago, Parsippany, NJ).

### ELISPOT assays

Enzyme-linked immunosorbent spot (ELISPOT) assays were performed to enumerate hF.IX-specific CD8^+^ T cells in mouse spleens, as previously described [3,24]. Briefly, splenocytes were plated at 1 × 10^6^ cells/well, and stimulated with media alone, staphylococcal enterotoxin B (Toxin Technologies, Sarasota, FL; 1 ug/mL), or the immunodominant CD8 epitope of hF.IX for the C3H-HeJ background (p74, Anaspec, San Jose, CA; 10 ug/mL) [3]. Analyses were performed in triplicate on individual mice. After stimulation for 20 hours, plates were harvested and IFN-γ spot-forming units (SFU) were detected and counted using the ImmunoSpot Analyzer (Cellular Technology, Shaker Heights, OH). Results were calculated as spot-forming units per 10^6^ total cells.

### Immunohistochemistry

Immunohistochemistry was performed using fluorescent antibodies on frozen and cryosectioned tissue, as previously described [25]. Briefly, muscle tissue was harvested and frozen in liquid N_2_-cooled 2-methylbutane. Cryosections (10 μm) of tissue were fixed in acetone at room temperature, blocked with 5% donkey serum (Sigma, St. Louis, MO), and stained with rat anti-CD8α (eBioscience, San Diego, CA) and goat anti-hF.IX (Affinity Biologicals, Ontario, Canada). Secondary antibody donkey anti-rat Alexa Fluor 488 and donkey anti-goat Alexa Fluor 568 (Life Technologies, Eugene, OR) were used for detection. Fluorescence microscopy was performed with a Nikon E800 microscope (Nikon, Tokyo, Japan).

### Statistics

Results are reported as means ± SEM. Significant differences between groups were determined with unpaired Student’s *t*-test. *P* values of <0.05 were considered significant. Analyses were performed using GraphPad Prism (San Diego, CA).

## Results

### The vector genome affects the CD8^+^ T cell response to F.IX in null mutation mice

To assess the effect of a scAAV genome on the immune response to F.IX, we injected hemophilia B (HB) C3H/HeJ mice intramuscularly (i.m.) with 10^11^ vector genomes (vg) of ss or scAAV serotype 1 vectors expressing human F.IX (hF.IX) under the control of a cytomegalovirus promoter (AAV1-CMV-hF.IX). These HB mice have a targeted deletion of the murine *F9* gene and therefore lack tolerance to F.IX antigen. In previous studies, we found that ssAAV2-CMV-hF.IX (serotype 2 vector) induced neutralizing antibody and CD8^+^ T cell responses against hF.IX upon i.m. injection in this strain [3]. Here, we used serotype 1 vector, because it is superior for muscle gene transfer and is hence in clinical trial/use for muscle gene transfer for α_1_-antitrypsin deficiency and for lipoprotein lipase deficiency [26-29].

**Figure 1:**
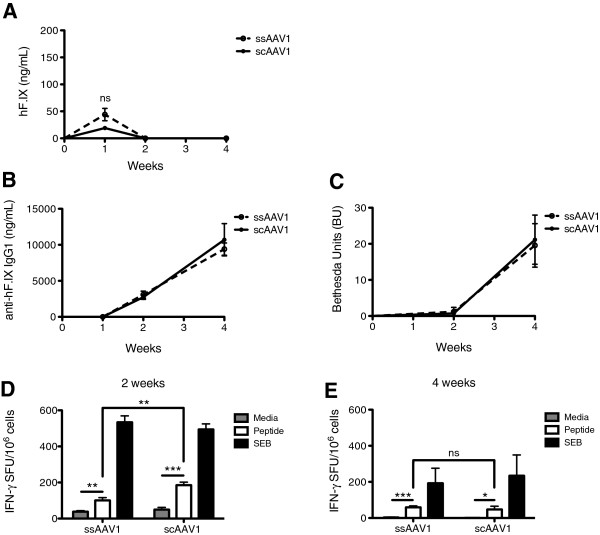
**Outcome of gene transfer with ss or scAAV1 in HB mice.** HB mice were injected i.m. with 10^11^ vg of ss or scAAV1-CMV-hF.IX (*n* = 4/group). Plasma was collected 1, 2, and 4 weeks post-injection. **(A)** Circulating hF.IX levels were measured by ELISA. **(B)** Anti-hF.IX IgG1 levels in plasma were measured by ELISA. **(C)** Bethesda titer. One BU represents the inhibition of 50% of clotting activity. **(D-E)** Splenocytes were harvested and restimulated with media alone, the CD8 epitope of hF.IX, or SEB, and IFN-γ spot-forming units (per 10^6^ cells) were measured by ELISPOT. Measurements were performed on individual animals two weeks **(D)** or four weeks **(E)** post-injection. Data points are averages ± SEM. Results are representative of at least two independent experiments. * P < 0.05, ** P < 0.01, *** P < 0.001, ns = not significant.

Plasma was then collected 1, 2, and 4 weeks post-injection to assess circulating expression of hF.IX as well as antibody responses to the transgene product. One week after vector injection, expression of hF.IX was detected in mice that received ss or scAAV1 (Figure 1A). At two weeks and thereafter, though, circulating hF.IX was not detected in either group of animals.

Corresponding with the loss of hF.IX expression in plasma, antibodies against hF.IX were first detected 2 weeks post-injection by ELISA (Figure 1B). Consistent with prior findings, these were of the IgG1 subclass, whereas levels of IgG2a and IgG2b were comparatively very low or nonexistent (data not shown) [3,30,31]. Average anti-hF.IX titers were nearly identical for both ss and scAAV vectors. To assess the functionality of this humoral immune response, we performed the Bethesda assay, which measures the ability of hF.IX-specific antibodies (inhibitors) to prevent plasma clotting activity. Inhibitor titers lagged behind the detection of anti-hF.IX IgG1, with no little or no inhibition of clotting detected after two weeks (Figure 1C). After 4 weeks, average titers of ~20 BU were measured regardless whether mice received ss or scAAV1.

**Figure 2:**
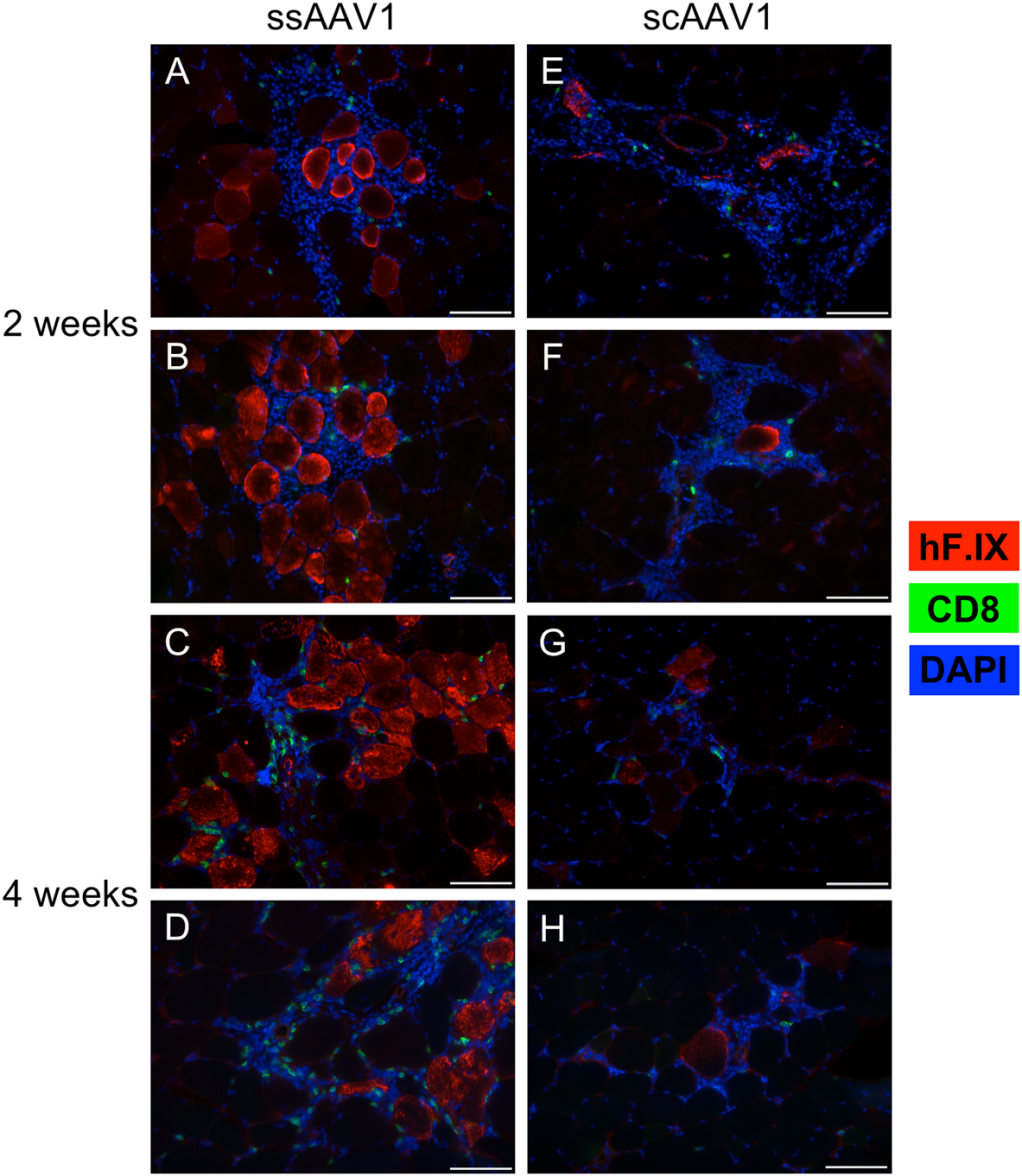
**Local hF.IX expression and CD8 infiltration in HB mice.** Skeletal muscle from HB mice injected i.m. with 10^11^ vg ss or scAAV1 (*n* = 4/group) was harvested, cryosectioned, and stained for hF.IX (red) and CD8 (green). Nuclei were visualized with DAPI (blue). Two weeks post-injection, tissue was analyzed from mice injected with ssAAV1 **(A-B)** or scAAV1 **(E-F)**. After four weeks, skeletal muscle was stained from mice injected with ssAAV1 **(C-D)** or scAAV1 **(G-H)**. Representative images from two mice are shown for each condition. The scale bar represents 100 μm. Results are representative of at least two independent experiments.

Two and four weeks post-injection, splenocytes were harvested to measure the CD8^+^ T cell response to hF.IX by ELISPOT. Both vectors induced a measurable antigen-specific response. However, mice that received scAAV1 had a significantly higher number of IFN-γ spot-forming units (SFU) when stimulated with the immunodominant CD8 epitope of hF.IX at 2 weeks (Figure 1D). Four weeks post-injection, all animals still showed a response, which was similar for ss and scAAV1-treated mice at this later time point (Figure 1E). Background SFU (media and SEB treatments) were higher at 2 weeks, possibly due to elevated immune activity at this time point. In order to assess whether activated hF.IX-specific CTLs infiltrated the transduced tissue, immunohistochemical analyses of injected muscles were performed. Two weeks post-injection, mice that received either ss or scAAV1 had significant CD8^+^ T cell infiltration, though there was more evidence of local hF.IX production in ssAAV1-treated mice (Figure 2A-B, E-F). At four weeks post-injection, muscle transduced with ssAAV1 maintained hF.IX expression concomitant with continued CD8^+^ T cell infiltrates, whereas mice that received scAAV1 had very few transduced skeletal muscle cells remaining, and CD8^+^ T cell infiltration had subsided (Figure 2C-D, G-H).

### Mice with a nonsense mutation fail to mount an immune response against F.IX regardless of the AAV genome

With the indication that scAAV vectors may induce a stronger CD8^+^ T cell response to hF.IX, we next sought to determine whether they could induce a response in hemophilic mice with a mutation that results in non-functional hF.IX expression. We had previously established hemophilic mice carrying *F9* missense mutations or a nonsense mutation. When injected i.m. with AAV2-CMV-hF.IX vector, none of the mice of either of these lines showed a CD8^+^ T cell response to F.IX; however, mice with a late stop codon mutation (at amino acid residue 338 of F.IX, “LS” line) produced antibodies against hF.IX, indicating that these mice were not fully tolerant to hF.IX [3]. Thus, we chose the LS line of hemophilic mice to test whether i.m. administration of an scAAV1 vector could break CD8^+^ T cell tolerance to hF.IX.

**Figure 3:**
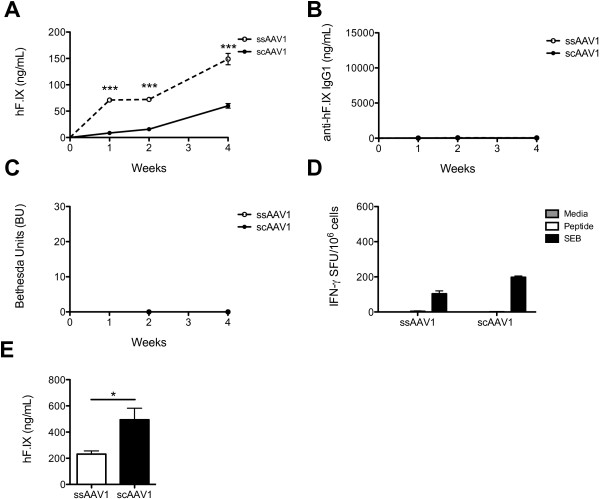
**Outcome of gene transfer with ss or scAAV1 in LS mice.** LS mice were injected i.m. with 10^11^ vg of ss or scAAV1-CMV-hF.IX (*n* = 4/group). Plasma was collected 1, 2, and 4 weeks post-injection. **(A)** Circulating hF.IX levels were measured by ELISA. **(B)** Anti-hF.IX IgG1 levels in plasma were measured by ELISA. **(C)** Bethesda titer. One BU represents the inhibition of 50% of clotting activity. **(D)** Splenocytes were harvested four weeks post-injection and restimulated with media alone, the CD8 epitope of hF.IX, or SEB, and IFN-γ spot-forming units (per 10^6^ cells) were measured by ELISPOT. Measurements were performed on individual animals. **(E)** Circulating hF.IX levels in C57BL/6 RAG^-/-^ mice 2 weeks post-injection with ss or scAAV1-CMV-hF.IX (*n* = 4/group). Data points are averages ± SEM. Results are representative of at least two independent experiments. * P < 0.05, *** P < 0.001, ns = not significant.

**Figure 4:**
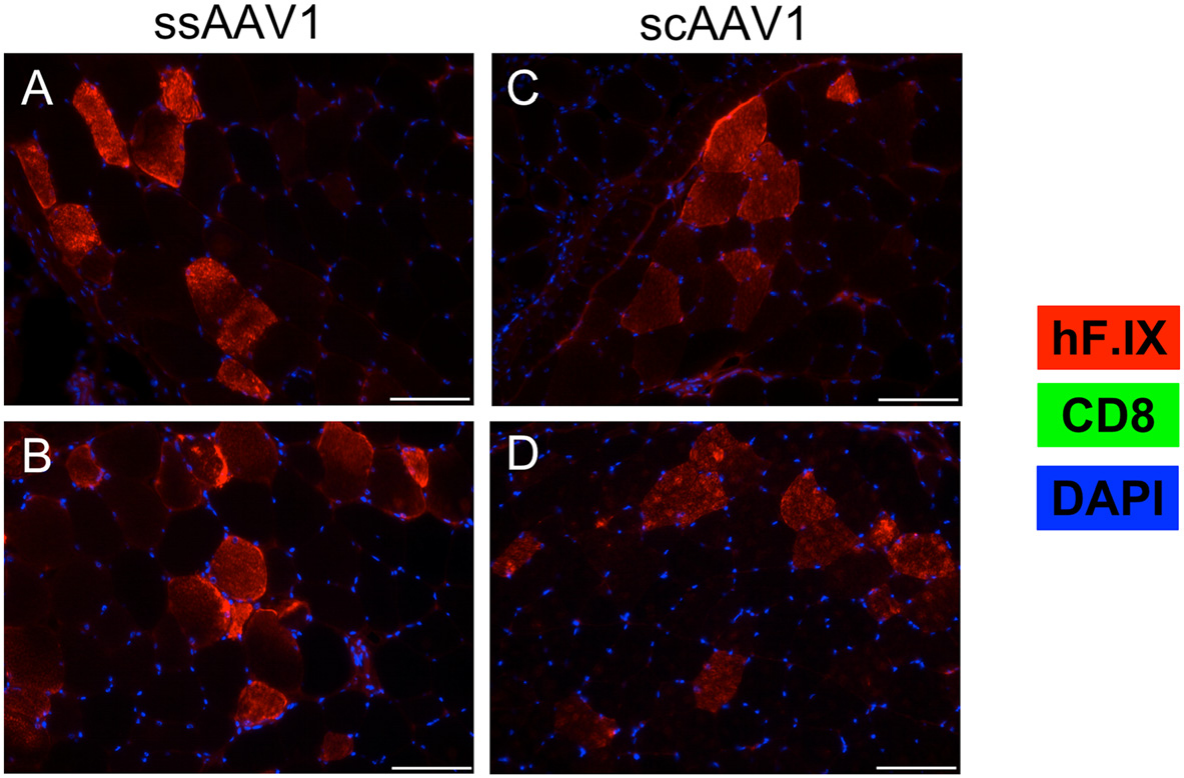
**Local hF.IX expression and CD8 infiltration in LS mice.** Skeletal muscle from LS mice injected i.m. with 10^11^ vg ss or scAAV1 (*n* = 4/group) was harvested, cryosectioned, and stained for hF.IX (red) and CD8 (green). Nuclei were visualized with DAPI (blue). Four weeks post-injection, tissue was harvested from mice injected with ssAAV1 **(A-B)** or scAAV1 **(C-D)**. Representative images from two mice are shown for each condition. The scale bar represents 100 μm. Results are representative of at least two independent experiments.

One week after gene transfer with either sc or ssAAV1 vectors, circulating hF.IX was detected at levels similar to those reported above for HB null mutation mice. At 2 and 4 weeks post-injection, hF.IX expression increased and persisted, with expression levels in ssAAV1-treated mice about 3-fold higher than scAAV1-injected mice after 4 weeks (Figure 3A). None of the LS mice developed antibodies/inhibitors against hF.IX over the course of the experiment (Figure 3B-C). After 4 weeks, splenocytes were once again harvested to measure the CD8^+^ T cell responses to hF.IX by ELISPOT. As with the humoral immune response, there was no evidence of splenic hF.IX-specific CD8^+^ T cells in LS mice treated with either vector (Figure 3D). The situation within the muscle itself reflected what had been observed systemically. Mice injected with either ss or scAAV1 showed similar transduction of skeletal muscle without evidence of infiltrating CD8^+^ T cells (Figure 4). In summary, use of scAAV vector did not increase the risk for humoral or cellular immune responses to the hF.IX transgene product in the context of the LS nonsense mutation.

Since LS mice displayed higher hF.IX expression levels from ssAAV1 vectors compared to scAAV1 in the absence of an immune response, we wanted to verify the functionality of the self-complementary vector on another background. Thus, RAG-deficient C57BL/6 mice that lack B and T cells were injected intramuscularly with 10^11^ vg of either vector. In these mice, circulating hF.IX levels were significantly higher in animals treated with scAAV1, suggesting that the inversion in expression levels observed in the LS mice may be a strain-specific effect (Figure 3E).

### Anti-capsid antibodies are not altered by scAAV vectors

**Figure 5:**
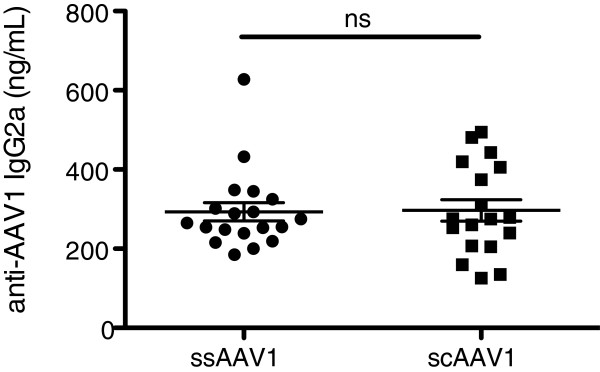
**Anti-capsid antibody response.** Plasma from HB or LS mice injected i.m. with 10^11^ vg of ss (*n* = 19) or scAAV1 (*n* = 18) was analyzed for the formation of anti-AAV1 IgG2a by ELISA 4 weeks post-injection. Data points represent individual mice, and the error bars show mean ± SEM. *ns = not significant.*

Finally, we investigated whether the vector genome may alter antibody responses against AAV capsid. Four weeks after i.m. injection of ss or scAAV1, we measured the formation of AAV1-specific antibodies (which are typically of a Th1 associated subclass such as IgG2a) in plasma by ELISA [13,32]. At this time point, levels of anti-AAV1 IgG2a were comparable whether mice received ss or scAAV1 (Figure 5). As with the transgene, capsid-specific antibody formation was not enhanced by scAAV vectors relative to ssAAV.

## Discussion

A major concern in gene replacement therapy is the potential for adaptive immune responses to the therapeutic transgene product, which may be recognized by the immune system as a foreign antigen. Our previous studies with hemophilic mice and dogs have clearly documented a major role for the underlying F.IX mutation on the risk of B and T cell responses to the transgene product in gene therapy for hemophilia B [3,17,23,33]. However, immune responses require activation signals, which may be derived from innate immune recognition of the vector. Hence, there are a number of additional factors that influence the likelihood, strength, and characteristics of an immune response. Among others, these include the choice and design of the vector, dose, and route of administration [4,21,34-38].

### Self-complementary vectors may increase immune responses to the transgene product depending on the route of vector administration

Self-complementary AAV vectors have been optimized for F.IX gene expression and have gathered growing enthusiasm because of the potential for improved gene transfer and expression [11,39,40]. At the same time, using scAAV instead of ssAAV may change innate immunity as well as the kinetics and magnitude of transgene expression. Here, we address how this change in vector genome conformation may influence immune responses to F.IX during muscle-directed gene transfer.

Innate immune responses to AAV vectors are typically weak and transient, resulting in limited inflammatory signals [13,41,42]. Nonetheless, we previously found that scAAV enhanced TLR9-dependent innate immune responses, resulting in stronger NF-κB dependent inflammation of tissue and expression of IFN I [13,43]. This increased immunogenicity, however, did not affect F.IX-specific immune responses and only modestly increased antibody formation against the vector in liver-directed gene transfer [13]. Hepatic transgene expression occurs in an environment characterized by active down-regulation of immune responses, thereby favoring induction of regulatory T cells and establishment of immune tolerance [8,44-49].

On the other hand, expression of a well-characterized vaccine antigen (HIV gag) in skeletal muscle yielded stronger and more functional CD8^+^ T cell responses, which was characterized by greater expression of cytokines and effector markers as well as increased lytic capability *in vivo*. Additionally, stronger antibody responses were observed when using scAAV compared to ssAAV vectors [50]. In hemophilia B mice with a *F9* gene deletion, we reconstituted some of these findings: the CD8^+^ T cell responses against hF.IX was more robust and also more functional using the scAAV vector, with infiltrating T cells rapidly eliminating hF.IX expressing muscle fibers. In the context of ssAAV gene transfer, the ensuing CD8^+^ T cell response results in chronic infiltration of transduced muscle without elimination of expression. These observations are consistent with out previous findings with ssAAV vectors [6]. CD8^+^ T cells induced by ssAAV have reduced cytotoxic and proliferative capacity that cannot be rescued by secondary immunization, most likely due to T cell exhaustion and apoptosis [50-52]. Additionally, it has been suggested that regulatory T cells induced by persistent AAV capsids in skeletal muscle were able to prevent elimination of transduced myocytes by chronically infiltrating CTLs in a clinical trial for α_1_-antitrypsin deficiency [27]. It is therefore possible that regulatory T cells could also be involved in our model. Although not addressed here, we previously found that administration of scAAV also increases CD8^+^ T cell responses to capsid compared to ssAAV [13].

In contrast, antibody responses against vector or transgene product seem less consistently affected by use of scAAV genomes. This may be explained by a greater dependence of CD8^+^ T cell responses than of antibody responses on TLR9 activation by AAV vectors [47,53]. Innate immune sensing of AAV vectors depends on TLR9 and is increased with scAAV due to increased TLR9 signaling from these vectors [13,15]. Interestingly, removal of CpG motifs from AAV vector genomes substantially reduces CD8^+^ T cell activation but has little effect on antibody formation [47]. Our results concur with these findings, as antibody responses to both transgene and capsid were not elevated with scAAV vectors.

### The underlying mutation is a greater determinant of the risk of immune responses to F.IX than the vector genome conformation

Previously, we bred hemophilia B mice onto the C3H/HeJ background, which gives higher antibody/inhibitor and CD8^+^ T cell responses to hF.IX than other common backgrounds. Mice with a null mutation (*F9* gene deletion) showed such responses to hF.IX in muscle gene transfer and suboptimal hepatic gene transfer [3,30,31,54]. These mice also form inhibitors and IgE responses during factor replacement therapy, resulting in anaphylaxis after repeated intravenous injections of F.IX protein [4,55]. However, optimal hepatic gene transfer with AAV vectors induces tolerance to hF.IX in this strain despite the gene deletion mutation [4,56,57]. Among the 3 other mutations that we examined (with endogenous non-functional hF.IX expression in hepatocytes; 2 missense and 1 nonsense mutation), the LS mutation (late stop codon) was the least tolerant and was still prone to antibody responses to hF.IX after muscle gene transfer using an ssAAV2 vector. Interestingly, no CD8^+^ T cell response was observed despite lack of expression of the C-terminus of hF.IX that contains the immunodominant CD8^+^ T cell epitope for this strain [3]. Given that our novel and published data demonstrated an increased ability of scAAV vectors to generate vigorous transgene product-specific CD8^+^ T cell responses, we hypothesized that a more potent scAAV1 vector may yield such a response in the LS strain. In spite of this, no CD8^+^ T cell response or antibody response was observed regardless of whether ss or scAAV1 vector was used. Together, results in null and LS mutations show that the underlying mutation is a stronger determining factor in the risk of immune responses to hF.IX than the type of AAV vector genome. The increased immunogenicity of the scAAV vector did not break tolerance to hF.IX in the LS mice, which do express the dominant CD4^+^ T cell epitope and may therefore exhibit tolerance in the T helper cell compartment. A comparison to our published data further suggests that use of AAV1 vector reduces antibody responses to hF.IX, at least in mice, when compared to AAV2 [3]. At least equally and perhaps more important than the underlying mutation is the route of vector administration/target tissue, with optimized hepatic gene transfer resulting in tolerance induction even for null mutations.

A somewhat curious result of the experiments in the tolerant LS strain were the higher levels of circulating hF.IX achieved with the ssAAV vector. Using the identical dose and vector preparations, scAAV vector outperformed ssAAV upon muscle gene transfer in immune deficient mice (RAG-deficient C57BL/6), which however were not available on a strain-matched C3H/HeJ genetic background. It is possible that the increased innate immune responses induced by scAAV vectors could be silencing expression of the transgene, which may be strain-specific. It is known that the activity of the CMV enhancer/promoter used in these vectors can be inhibited by inflammatory cytokines [58,59]. IL-12-mediated inflammation at the time of gene transfer has also been shown to inhibit transgene production [60]. Similarly, the expression of HIV gag p24 and induction of gag-specific CD8^+^ T cells was previously shown to be lower at a dose of 10^11^ than 10^10^ vg, a phenomenon which may have also been related to silencing of the CMV promoter, or saturation of the transduction capacity of the injected muscle at a dose of 10^10^ vg [50]. Although we previously found that IFN I induced by recombinant adenovirus but not by scAAV caused transgene silencing, a transthyretin rather than a CMV promoter was used in the scAAV vectors in that study [61]. Clearly, there are still factors affecting transgene expression from scAAV vectors that remain to be elucidated.

## Conclusion

In summary, when performing gene transfer with AAV vectors via a route of administration that is more prone to immune responses to the transgene product, the underlying genetic defect is an important determinant of the risk of B and T cell responses. Should an immune response ensue, which may be more likely to occur when treating in the context of a null mutation, scAAV vectors are likely to cause a more potent CD8^+^ T cell response than ssAAV, thereby increasing the risk of loss of transduced cells. These observations likely apply to gene therapies for other genetic diseases and should be taken into consideration during clinical trial design.

## Abbreviations

hF.IX: Human factor IX; F9: Factor IX; AAV: Adeno-associated virus; ssAAV: Single-stranded AAV; scAAV: Self-complementary AAV; CTL: Cytotoxic T lymphocyte; HB mice: Hemophilia B null-mutation mice; LS: Late-stop codon hemophilic mice; BU: Bethesda unit; RAG: Recombination-activating gene; ELISA: Enzyme-linked immunosorbent assay; ELISPOT: Enzyme-linked immunosorbent spot assay; DAPI: 4′,6-diamidino-2-phenylindole.

## Competing interests

RWH has been receiving royalty payments from Genzyme Corp. for license of AAV-FIX technology.

## Authors’ contributions

GLR, ATM, and IZ performed experiments. GLR, ATM, HCE, and RWH designed experiments. GLR, ATM, HCE, and RWH interpreted data. HCE and RWH supervised and coordinated the study. GLR, HCE, and RWH wrote the manuscript. All authors read and approved the final manuscript.
